# Association Between Changes in Postpartum Weight and Waist Circumference and Changes in Cardiometabolic Risk Factors Among Women With Recent Gestational Diabetes

**DOI:** 10.5888/pcd16.180308

**Published:** 2019-04-18

**Authors:** Jacinda M. Nicklas, Bernard A. Rosner, Chloe A. Zera, Ellen W. Seely

**Affiliations:** 1 Division of Endocrinology, Diabetes and Hypertension, Brigham and Women's Hospital, Boston, Massachusetts; 2 Division of General Medicine and Primary Care, Beth Israel Deaconess Medical Center, Boston, Massachusetts; 3 Division of General Internal Medicine, University of Colorado School of Medicine, Aurora, Colorado; 4 Channing Division of Network Medicine, Department of Medicine, Harvard Medical School, Boston, Massachusetts; 5 Department of Biostatistics, Harvard School of Public Health, Boston, Massachusetts; 6 Division of Maternal-Fetal Medicine, Department of Obstetrics and Gynecology, Brigham and Women's Hospital Boston, Massachusetts

## Abstract

**Introduction:**

Women with gestational diabetes mellitus (GDM) have a 30% to 70% risk for developing type 2 diabetes and are at increased risk for cardiovascular disease. Little is known about how anthropometric changes in the first postpartum year modify cardiometabolic risk factors.

**Methods:**

We randomly assigned women in the Balance After Baby study to an intervention group consisting of participation in a web-based lifestyle program or to a control group in which no program was offered. We measured weight, height, waist circumference, blood pressure, lipids, insulin, adiponectin, interleukin-6, and high-sensitivity C-reactive protein, and we conducted 2-hour oral glucose tolerance tests at 6 weeks, 6 months, and 12 months postpartum. We evaluated whether women assigned to the intervention had improved cardiometabolic risk markers compared with the control group. We then conducted a post-hoc analysis, pooling the 2 groups to compare changes in weight and waist circumference with changes in cardiometabolic risk factors.

**Results:**

Women in the intervention group did not significantly improve cardiometabolic risk markers compared with women in the control group. We noted a large overlap of weight change and change in waist circumference between groups. In our post-hoc analysis pooling groups, changes in diabetes and cardiovascular risk factors were significantly correlated with changes in weight and waist circumference. The strongest associations were observed for fasting insulin, HOMA, and fasting glucose.

**Conclusion:**

Anthropometric changes in weight and waist circumference in women with recent GDM may affect cardiometabolic risk factors, even in the first postpartum year. Our study demonstrates the importance of the postpartum year as an opportunity to decrease future risk for diabetes and cardiovascular disease in women with a history of GDM.

**Summary**

**What is already known on this topic?**

Several longer term studies demonstrated that weight loss decreases the risk for conversion to type 2 diabetes in women with a history of gestational diabetes.

**What is added by this report?**

This report shows that changes in weight and waist circumference in the postpartum year among women with recent GDM are significantly correlated with changes in cardiometabolic risk factors.

**What are the implications for public health practice?**

This study adds further evidence to the potential for the postpartum year to serve as a window of opportunity to decrease future risk of cardiometabolic disease.

## Introduction

The incidence of gestational diabetes mellitus (GDM) in US women is from 3% to 9%, depending on the population and the diagnostic criteria used (1). The incidence of GDM has been rising with the increasing prevalence of overweight and obesity in the United States. Women with a history of GDM have an increased risk of developing type 2 diabetes (2), with a rapid increase in cumulative risk within the first 5 years postpartum (3) and an elevated risk sustained through 10 years postpartum (4). Type 2 diabetes is also a key risk factor for cardiovascular disease (CVD) in women because of its higher relative and absolute association with CVD in women compared with men (5). Studies show an increased 10-year CVD risk in women with GDM history (6), with evidence of earlier incidence of atherosclerosis (7) and a 26% increased risk of developing hypertension over 16 years (8). 

In 2011 the American Heart Association published updated guidelines to include a history of GDM as a CVD risk factor when screening parous women (9), and they recommend surveillance and management of CVD risk factors in women with a history of GDM. Despite these risks, little prospective research has examined the effects of postpartum weight changes or changes in waist circumference on cardiometabolic risk following pregnancies complicated by GDM (10). Although several intervention trials have demonstrated significant reductions in weight among postpartum women with recent GDM, it is unclear how clinically significant the weight loss is, and they have not clearly demonstrated improvements in blood glucose levels or other cardiometabolic risk factors (11).

In the Balance after Baby study we adapted the Diabetes Prevention Program into a web-based lifestyle intervention designed to decrease postpartum weight retention in women with recent GDM (12). Women randomly assigned to the intervention group watched instructional videos, worked with a lifestyle coach, and were asked to track their weight and steps. In the analysis of the primary outcome, women assigned to the intervention group lost significantly more weight than women randomized to the control group (12). However, women randomly assigned to the intervention group did not demonstrate a significant improvement in glucose tolerance when compared with the control group, possibly because of the short follow-up period and small sample size (N = 75).

For this analysis we first sought to determine the effect of the web-based lifestyle intervention program on markers of cardiometabolic risk by analyzing the change by group assignment. Given the small sample size and substantial overlap in change in postpartum weight and waist circumference, we then pooled the sample to conduct post-hoc analyses to explore the effect of postpartum weight loss and changes in waist circumference on markers of cardiometabolic risk, irrespective of study group assignment. We sought to determine whether anthropometric changes in the first postpartum year were significantly associated with changes in cardiovascular, metabolic, and inflammatory markers.

## Methods

### Study population

The Balance after Baby randomized controlled trial (no. NCT01158131) was a prospective intervention study of 75 women with recent GDM to examine the effect of a web-based lifestyle intervention on postpartum weight loss. Feasibility and efficacy of the trial have been reported (12). Briefly, 75 postpartum women were studied in the first year after a pregnancy complicated by GDM. The study was conducted at Brigham and Women’s Hospital in Boston, Massachusetts, from June 2010 through September 2012. Inclusion criteria were being aged 18 to 45; having a diagnosis of GDM in the most recent pregnancy, according to Carpenter-Coustan criteria (13) or by documented clinical diagnosis in the medical record; and having a body mass index (BMI, kg/m^2^) of 24 or higher (≥22 for Asian participants) and less than 50. Women were excluded if they had a history of type 2 diabetes or bariatric surgery or if they were taking medications known to affect glucose metabolism or weight. Women were also excluded if they delivered before 32 weeks gestation or if they had net weight loss during pregnancy.

Qualifying participants attended an initial study visit at approximately 6 weeks postpartum for baseline measurements, and measures conducted at baseline were repeated at approximately 6 months and approximately 12 months postpartum. Women diagnosed with type 2 diabetes (by 2 abnormal oral glucose tolerance test [OGTT] values or a single abnormal value repeated and again found to be abnormal) at the 6-month visit completed the 12-month study visit excluding the OGTT. For the pooled analysis, we restricted the sample to women who had baseline measurements of anthropometry, blood pressure, and biomarkers, as well as anthropometric measurements, blood pressure, and a fasting blood sample at 6 months and/or 12 months.

### Measurements

We measured weight, height, and waist circumference, and we also measured markers of cardiometabolic risk: hemoglobin A1c (HbA_1c_), fasting glucose, 75 g 2-hour OGTTs, fasting insulin, adiponectin, low-density lipoprotein (LDL) cholesterol, high-density lipoprotein (HDL) cholesterol, fasting triglycerides, blood pressure, and the inflammatory markers high-sensitivity C-reactive protein (hsCRP) and interleukin-6 (IL-6). We requested that women not perform exercise nor breastfeed during the OGTT. We calculated BMI and HOMA-IR ([fasting glucose × fasting insulin)/405).

### Statistical analysis

We compared baseline characteristics using *t* tests or Wilcoxon rank sum tests for continuous variables and Pearson’s χ^2^ or Fisher’s exact tests for categorical variables. We calculated means for data that were normally distributed and medians for skewed data. Women who became pregnant, started assisted reproductive treatment, or had a neonatal death were censored at the time of these events. We estimated mixed-effects regression models using a random intercept and an unstructured covariance matrix to look at the effect of group assignment on change in markers of cardiometabolic risk in the intent-to-treat sample (n = 75). We controlled for baseline values of the cardiometabolic risk factors in the mixed-effects regression models. Data after censoring events were considered missing at random, except that we carried forward blood glucose and HbA_1c_ values for 2 women who were diagnosed with type 2 diabetes at 6 months. As a sensitivity analysis, we estimated the mixed-effects regression models while controlling for breastfeeding status. For the post-hoc analysis of the pooled sample, we estimated regression models to assess the effect of changes in weight and waist circumference on markers of cardiometabolic risk. We controlled for differences at baseline by including the variable for the risk factor at the baseline visit in each model. We conducted a sensitivity analysis, controlling for breastfeeding status in each model. We used JMP 12–13 Pro SAS statistical software and SAS version 9.0 to conduct analyses (SAS Institute, Inc).

## Results

Participants had a mean age of 33 years and a mean baseline postpartum BMI of 31 (Table 1). Fifty-seven percent were white, 31% were African American, 12% were Asian, and 20% identified as Hispanic. The only significant difference in characteristics between the intervention and control groups at baseline was diastolic blood pressure. 

**Table 1:** Characteristics of Intervention and Control Group Participants (N = 75), at 6-Week Postpartum Baseline Visit, Balance After Baby Randomized Trial, Boston, Massachusetts, June 2010–September 2012^a^

Characteristic	Intervention Arm (n = 36)	Control Arm (n = 39)	*P* Value
Age, y	33.6 (4.8)	33.2 (5.8)	.75
Race, no. (%)			
White	23 (64)	20 (51)	.57
African American	9 (25)	14 (36)	
Asian	4 (11)	5 (13)	
Hispanic/Latina	9 (25)	6 (15)	.39
Prepregnancy weight, kg	80.7 (18.7)	81.2 (18.3)	.90
Prepregnancy BMI, kg/m^2^	30.3 (6.3)	30.5 (5.7)	.89
Gestational weight gain, kg	11.7 (8.0)	13.2 (7.6)	.42
Weeks postpartum at baseline visit	7.3 (2.6)	7.0 (1.6)	.53
Breastfeeding (exclusively or mixed), no. (%)	27 (75)	30 (77)	.85
Baseline postpartum weight, kg	82.9 (17.3)	84.2 (19.0)	.75
Baseline postpartum BMI, kg/m^2^	31.2 (5.8)	31.6 (5.5)	.77
Baseline waist circumference, cm	100.1 (14.2)	102.2 (15.0)	.52
Fasting blood glucose, mg/dl, median (IQR)	91 (86–98)	92 (88–96)	.82
Glucose at 120 min, mg/dL, median (IQR)	122.5 (95–138)	125 (103–139)	.75
HbA_1c_	5.7 (0.4)	5.9 (0.3)	.08
Fasting insulin, uU/ml, median (IQR)	6.1 (4.0–7.9)	6.7 (4.0–8.3)	.71
HOMA, median (IQR)	1.4 (0.90–1.9)	1.4 (0.88–2.1)	.65
Adiponectin	4.0 (1.9)	3.9 (1.2)	.70
Low-density lipoprotein cholesterol, mg/dL	122 (30)	129 (35)	.58
High-density lipoprotein cholesterol, mg/dL	55 (13)	55 (14)	.96
Triglycerides, median (mg/dL)	94 (62–118)	109 (73–136)	.16
Systolic blood pressure, mm Hg	125 (15)	120 (13)	.24
Diastolic blood pressure, mm Hg	84 (9)	79 (9)	.037
hsCRP, median (IQR)	2.4 (1.0–5.9)	3.7 (1.8–5.8)	.20
IL-6, median (IQR)	2.2 (1.7–3.5)	2.8 (1.7–3.6)	.57

Abbreviations: BMI, body mass index; HbA_1c_, hemoglobin A_1c_; HOMA, homeostatic model assessment; HsCRP, C-reactive protein; IL-6, interleukin 6; IQR, interquartile range; SD, standard deviation.

a Values are mean (SD) unless otherwise indicated.

**Figure 1:**
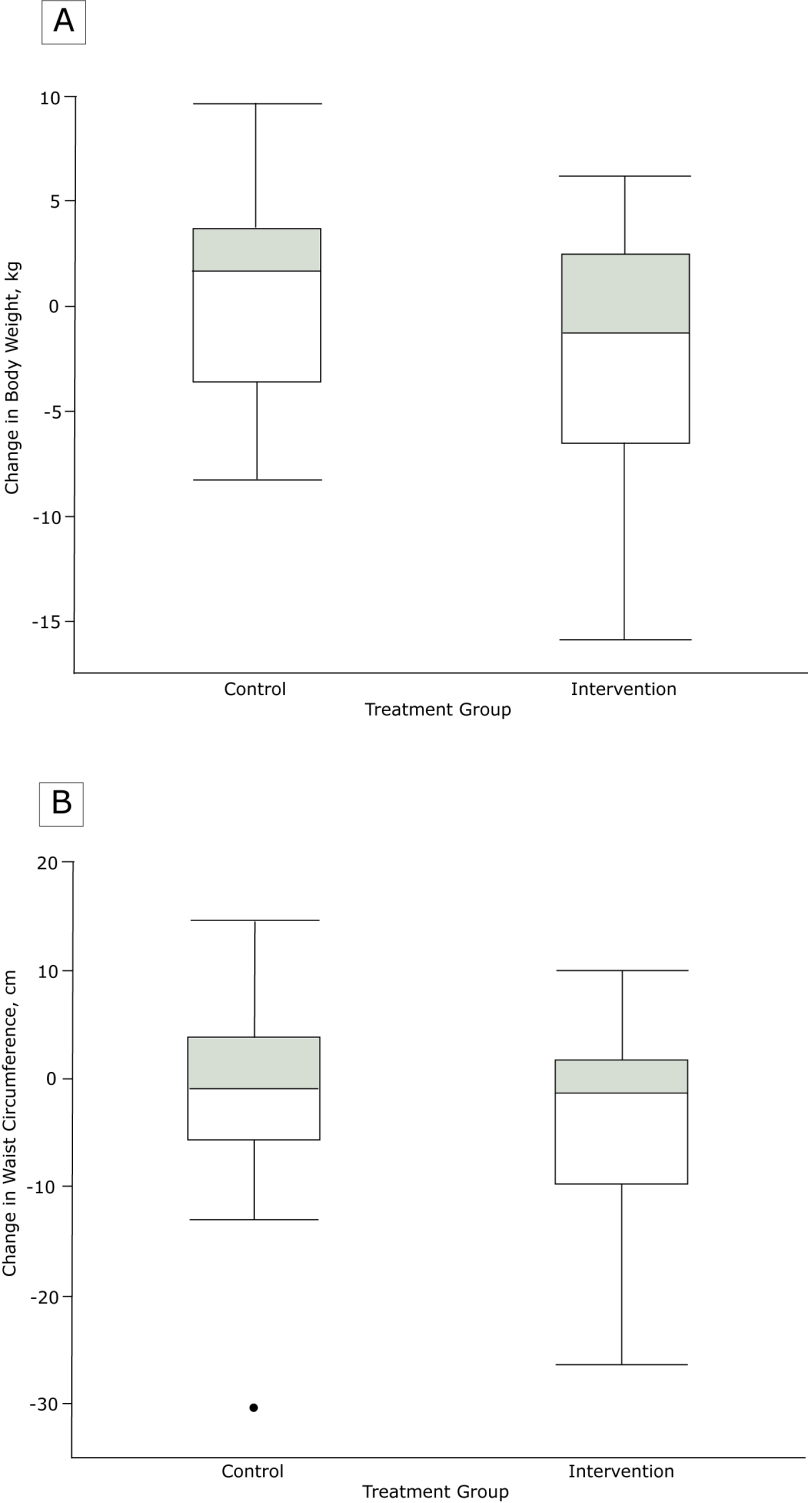
Distribution of change in a) weight and b) waist circumference in the control and intervention groups at 12 months postpartum.

Women assigned to the intervention group had lost significantly more weight than those assigned to the control group at 6 months (−2.6 kg [95% confidence interval (CI), −4.4 to −0.8] vs 1.4 kg [95% CI, −0.4 to 3.1]; *P* = .003) and at 12 months (−2.8 kg [95% CI, −4.8 to −0.7] vs 0.5 kg [95% CI, −1.4 to 2.4]; *P* = .02 for difference between groups) (Table 2). We noted overlap in the intervention and control groups for both change in weight (−15.9 kg to 9.6 kg) and change in waist circumference (−30.6 cm to 14.5 cm) (Figure 1). The women assigned to the intervention group also had a significant decrease in BMI compared with the control group. Similarly, women in the intervention group were closer to their prepregnancy weight (−0.7 kg; 95% CI, −3.5 to 2.2) than women in the control group (4.0 kg, 95% CI, 1.3 to 6.8). More women in the intervention group with measured weights (11 of 28, 39%) compared with the control group (7 of 32, 22%) achieved weight loss of greater than or equal to 5%.

**Table 2:** Change in Cardiometabolic Risk Factors in Intervention and Control Groups Among Postpartum Women (N = 75), Boston, Massachusetts, June 2010–September 2012

Type of Risk Factor	Change at 6 Months Postpartum			Change at 12 Months Postpartum		
	Control	Intervention	*P *Value^a^	Control	Intervention	*P *Value^a^
	Estimated Mean or % Change From Baseline 95% CI			Estimated Mean or % Change From Baseline 95% CI		
**Cardiometabolic**						
Weight, kg	1.4 (−0.4 to 3.1)	−2.6 (−4.4 to −0.8)	.003	0.5 (−1.4 to 2.4)	−2.8 (−4.8 to −0.7)	.02
BMI, kg/m^2^	0.5 (−0.2 to 1.2)	−1.0 (−1.7 to −0.3)	.002	0.2 (−0.5 to 0.9)	−1.1 (−1.9 to −0.4)	.03
Waist circumference, cm	−0.7 (−3.5 to 2.0)	−3.3 (−6.1 to −0.5)	.20	−1.0 (−4.0 to 2.1)	−4.2 (−7.4 to −1.0)	.15
**Diabetes-related**						
Fasting glucose, % change	5.3 (1.0 to 9.6)	2.8 (−1.4 to 7.3)	.43	5.9 (1.8 to 10.3)	5.5 (1.2 to 10.1)	.89
Glucose at 120 min, % change	8.8 (−0.3 to 18.6)	6.3 (−2.9 to 16.4)	.72	−6.3 (−15.9 to 4.4)	−2.7 (−13.2 to 9.0)	.64
HbA_1c_, %	0.0 (−0.2 to 0.1)	0.0 (−0.1 to 0.1)	.82	0.0 (−0.1 to 0.1)	0.0 (−0.2 to 0.1)	.98
Fasting insulin, % change	12.8 (−8.7 to 39.4)	6.0 (−14.8 to 32.0)	.69	26.5 (3.7 to 54.4)	32.3 (7.1 to 63.4)	.76
HOMA, % change	17.9 (−7.0 to 49.5)	8.5 (−15.1 to 38.7)	.63	35.4 (8.2 to 69.5)	40 (10.6 to 78.1)	.83
Adiponectin	−0.1 (−0.5 to 0.4)	0.1 (−0.4 to 0.6)	.59	0 (−0.3 to 0.4)	0.1 (−0.3 to 0.5)	.74
**Cardiovascular**						
LDL cholesterol, mg/dl	−19.2 (−27.2 to −11.2)	−18.4 (−26.9 to −9.9)	.89	−21.0 (−29.7 to −12.3)	−18.8 (−28.3 to −9.2)	.73
HDL cholesterol, mg/dl	−3.0 (−6.1 to 0)	−4.2 (−7.4 to −1.0)	.61	−3.9 (−7.2 to −0.5)	−5.5 (−9.2 to −1.9)	.51
Triglycerides, mg/dl	−12.6 (−24 to 1)	−9.0 (−21.9 to 6.0)	.70	−11.8 (−23.9 to 2.2)	−1.2 (−15.8 to 15.9)	.30
SBP, mm Hg	−1.2 (−5.0 to 2.7)	−3.6 (−7.6 to 0.5)	.39	−2.4 (−6.9 to 2.1)	−3.9 (−8.8 to 1.0)	.66
DSP, mm Hg	−2.3 (−5.3 to 0.8)	−4.1 (−7.2 to −0.9)	.42	−5.0 (−7.6 to −2.3)	−6.5 (−9.4 to −3.6)	.45
**Inflammatory markers**						
hsCRP, % change	−30.1 (−50.1 to −3.9)	−14.0 (−38.8 to 21.1)	.36	−33.9 (−50.0 to −12.6)	−41.3 (−56.6 to −20.4)	.57
IL6, % change	−10.1 (−31.3 to 17.7)	0.2 (−24.6 to 33.3)	.58	−8.6 (−25.1 to 11.4)	−23.7 (−38.7 to −5.1)	.23

Abbreviations: BMI, body mass index; CI, confidence interval; DSP, diastolic blood pressure; HbA_1c_, hemoglobin A_1c_; HDL, high-density lipoprotein; HOMA, homeostatic model assessment; hsCRP, C-reactive protein; IL-6, interleukin 6; IQR, interquartile range; LDL, low-density lipoprotein; SBP, systolic blood pressure; SD, standard deviation.

a *P* value for change between groups.

We observed no significant difference in change in waist circumference between women assigned to the intervention group and women assigned to the control group, although it trended in the same direction as weight and BMI at both 6 and 12 months. Overall fasting insulin and HOMA-IR tended to increase over time in both groups, and fasting lipids decreased over time in both groups. HsCRP also decreased in both groups over time. In the sensitivity analysis, including a variable in the mixed-effects regression models to represent breastfeeding status over time did not substantively change the findings.

**Figure 2:**
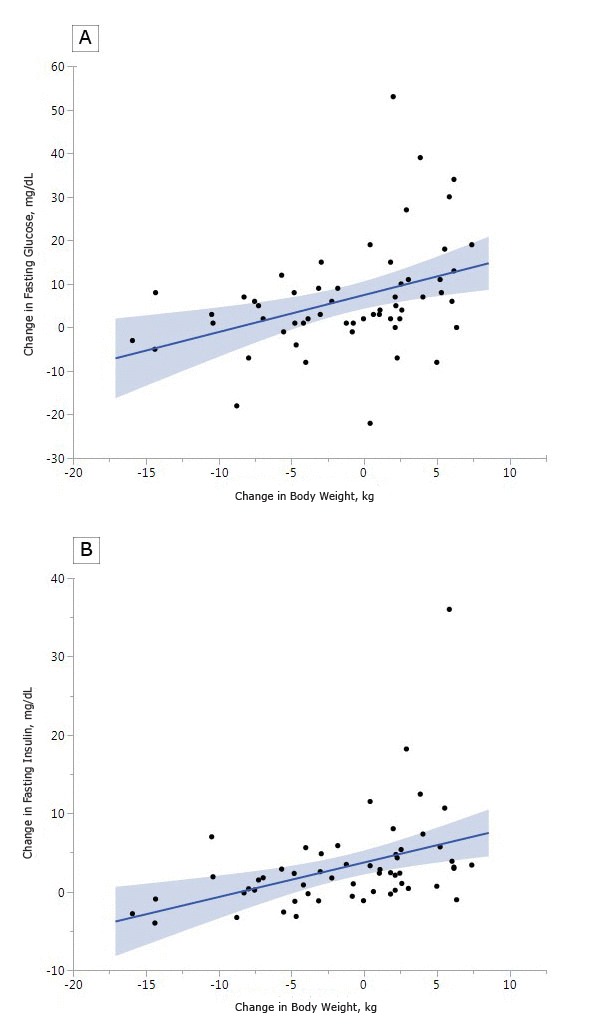
Change in a) fasting glucose vs. change in weight at 12 months postpartum and b) fasting insulin and change in weight at 12 months postpartum.

In the pooled analysis, irrespective of group assignment, a positive change in weight was significantly associated with changes in most diabetes risk markers (Table 3). Some of the strongest relationships were seen for the change in fasting glucose and fasting insulin (Figure 2). The difference in mean fasting glucose between the tenth percentile of weight change and the 90th percentile of weight change in the entire cohort at 6 months (15.3 kg) was 12.5%. Over the 10th to 90th percentile range at 12 months (14.7 kg) it was 12.1% over the 12 month range. For fasting insulin, the change over the 10th to 90th percentile of weight change was 48% for 6 months and 58% at 12 months. 

**Table 3:** Association Between Change in Weight and Waist Circumference and Markers of Cardiometabolic Risk of Participants (N = 75), Balance After Baby Randomized Trial, Boston, Massachusetts, June 2010–September 2012^a^

Cardiometabolic Risk Factor	Change in Weight						Change in Waist Circumference					
	6 month			12 month			6 month			12 month		
	β	Change Over Range of +15.3 kg	*P*	β	Change Over Range of +14.7 kg	*P*	β	Change Over Range +18.0 cm	*P*	β	Change Over Range +24.1 cm	*P*
Fasting glucose, % change	0.00869	12.5	<.001	0.00875	12.1	.002	0.00724	12.2	<.001	0.00452	10.3	.03
Glucose at 120 min, % change	0.01313	18.2	.007	0.00101	1.5	.88	0.00617	10.5	.13	0.00681	15.1	.13
HbA_1c_, % change	0.01967	0.3	.024	0.02542	0.4	<.001	0.01407	0.3	.06	0.01878	0.5	<.001
Fasting insulin, % change	0.04276	48.1	<.001	0.05037	58.3	<.001	0.04234	53.0	<.001	0.03474	56.7	<.001
HOMA, % change	0.05201	55.0	<.001	0.05939	57.8	<.001	0.05082	60.0	<.001	0.03937	61.3	<.001
Adiponectin, ng/L	−0.04327	−0.7	.14	−0.07633	−1.1	<.001	−0.01561	−0.28	.54	−0.04421	−1.1	.007
LDL cholesterol, mg/dL	0.84853	13.0	.09	0.59048	8.7	.28	0.74374	13.3	.09	0.48164	11.6	.21
HDL cholesterol, mg/dL	−0.05576	−0.9	.76	−0.39357	−5.8	.05	−0.17434	−3.1	.26	−0.22598	−5.4	.13
Triglycerides, % change	0.02264	29.3	.007	0.02828	34.1	.002	0.01343	21.4	.07	0.00525	11.9	.45
SBP, mm Hg	0.27110	4.1	.18	0.31292	4.6	.26	0.32090	5.8	.08	0.27254	6.6	.16
DSP, mm Hg	0.22410	3.4	.21	0.26673	3.9	.13	0.11389	2.0	.46	0.11124	2.7	.38
hsCRP, change over range	0.02949	36.4	.17	0.04263	46.6	.02	0.02277	33.6	.21	0.04121	62.9	.001
IL-6, change over range	0.00127	1.9	.93	0.00343	4.9	.78	0.02040	30.7	.10	0.00651	14.5	.46

Abbreviations: DSP, diastolic blood pressure; HbA_1c_, hemoglobin A_1c_; HDL, high-density lipoprotein; HOMA, homeostatic model assessment; hsCRP, C-reactive protein; IL-6, interleukin 6; LDL, low-density lipoprotein; SBP, systolic blood pressure.

a Estimates shown over the 10th to 90th percentile range for weight change.

Change in waist circumference was also significantly correlated with most diabetes risk markers, including fasting glucose and fasting insulin (Table 3). A change in triglycerides was significantly associated with weight change at both 6 and 12 months but not with change in waist circumference; hsCRP was significantly associated with both weight change and change in waist circumference at 12 months postpartum. None of the values changed significantly in sensitivity analyses when controlling for breastfeeding status. A substantial proportion of participants had 120-minute glucose values that were lower than their fasting glucose values (13% at 6 weeks, 9% at 6 months, and 24% at 12 months).

## Discussion

We noted significant changes between the intervention and control groups for change in weight between baseline and 6 and 12 months postpartum, as well as the change from prepregnancy weight. In this population of women with recent GDM, a return to prepregnancy weight is clinically significant with respect to the prevention of type 2 diabetes and cardiovascular disease (12). However, women assigned to the intervention group did not have a significant improvement in markers of cardiometabolic risk when compared with the control group. This may have been because of the small sample size, the duration of the study, and the overlap in change in weight in both groups. In our post-hoc analysis in which the 2 groups were pooled, we noted that changes in diabetes and cardiovascular risk factors were significantly correlated with changes in both weight and waist circumference. 

Overall, changes in weight were more often significantly correlated with cardiometabolic risk factors than changes in waist circumference. The strongest associations were seen for fasting insulin, HOMA, and fasting glucose. These findings illustrate that changes in weight and waist circumference in women with recent GDM have a significant relationship with diabetes risk factors, even in the first postpartum year. Although risk factors for cardiovascular disease had fewer significant correlations, significant relationships were noted between triglycerides and change in weight, as well as changes in weight and waist circumference and change in hsCRP.

Our findings demonstrated the strongest relationships between change in weight and markers of insulin sensitivity, including fasting glucose and insulin. The impact of weight loss on insulin sensitivity is well established. Studies demonstrate that postpartum insulin sensitivity is one of the strongest predictors of progression to type 2 diabetes following GDM in the first 5 years following the index pregnancy (14). Other studies looking at longer-term outcomes in women with prior GDM have shown the impact of weight changes on the incidence of type 2 diabetes. In one prospective cohort study of 1,695 women with a history of GDM, increases in weight and BMI were associated with increased risk of type 2 diabetes over 18 years of follow-up (15). Similarly, a retrospective cohort study of 1,263 Chinese women within 5 years of a GDM delivery demonstrated significantly increased risk for type 2 diabetes and prediabetes with weight gain and, conversely, decreased risk with weight loss (16). Post-hoc analyses of the Diabetes Prevention Program demonstrated that among older women who were on average 12 years away from their GDM pregnancy, women assigned to an intensive lifestyle program developed type 2 diabetes at a rate 50% lower than those women who had been assigned to the control group (17). These findings support recommendations in the literature suggesting that weight loss in the first postpartum year should delay or prevent onset of type 2 diabetes. 

We did not find a significant association between 2-hour glucose and weight change in the pooled analysis. This may be because there was a substantial percentage of women with 2-hour glucose values lower than their baseline fasting glucose values. It has been hypothesized that these paradoxically low glucose values may be a precursor to insulin resistance (18). This may also be related to exercise, which was not objectively measured in this study.

Markers of cardiovascular risk have been less studied in the early postpartum period among women with a history of GDM. In one prospective study of 305 women, of whom approximately 30% had GDM, Kew and colleagues reported that women who gained weight between 3 and 12 months postpartum tended to have worsening of certain cardiometabolic risk factors, including blood pressure, HOMA-IR, adiponectin, and LDL cholesterol (19). They did not see differences in OGTT results, triglycerides, HDL cholesterol or hsCRP after adjustment for covariates, and they did not evaluate changes in waist circumference. Given that the adverse metabolic profiles were largely not apparent at 3 months but emerged at 12 months, the authors suggested increased focus on the postpartum year for limiting postpartum weight retention (19) through interventions and increasing physical activity. In our study we saw a pronounced decrease in both lipids and hsCRP from baseline to 12 months. Lipid levels are known to increase during pregnancy and return to baseline postpartum (20). In our study, among cardiovascular risk factors, only triglycerides demonstrated a significant relationship with weight changes in the postpartum year. Study findings are mixed for hsCRP levels in pregnancy and postpartum; some showed an increase through pregnancy and postpartum and others demonstrated a decrease. Studies demonstrated larger increases among obese women (21,22). In our study, we noted an expected decrease in hsCRP overall postpartum, and a significant relationship with changes in weight at 12 months.

Fewer data are available on the relationship between changes in waist circumference and cardiometabolic risk factors. Among studies in general populations, findings are mixed on whether changes in weight or waist circumference are a more powerful predictor of changes in cardiometabolic risk factors (23–26). Although decreases in waist circumference improve triglycerides, blood pressure, and hyperglycemia in nonpostpartum populations (27), little is known about these changes in the postpartum period. Given the changes in waist circumference related to childbearing as well as parity, the relationship between changes in waist circumference and cardiometabolic risk factors may be more difficult to characterize. Several studies have demonstrated that postpartum weight retention tends to be more centrally distributed, which likely increases the likelihood of worsening cardiometabolic risk (28). One long-term study (28) with a mean follow-up of 7.3 years noted that postpartum increases in weight and waist circumference among Finnish women with GDM during pregnancy predicted the development of hyperglycemic conditions (10).

Limitations of this study include its being a single-site study with a small sample size and the use of multiple correlations to analyze the relationships between weight change and cardiometabolic markers. The pooled analysis was a post-hoc analysis and therefore should be considered hypothesis-generating. Our data set also contained missing values at some points.

Studies show that pregnancy weight retained beyond 6 to 12 months postpartum is more likely to be retained long-term and is a powerful independent risk factor for future obesity (29), which has prompted calls for decreasing postpartum weight retention. The findings from our analysis provide further justification for early postpartum weight loss and the reduction of waist circumference given the association with changes in cardiometabolic risk factors even during this first year after delivery. Our study adds further evidence to the potential for the postpartum year to serve as a window of opportunity to decrease future risk of obesity and chronic disease (30), particularly diabetes. Future studies should also examine how changes in these cardiometabolic risk factors relate to the acquisition of diabetes and cardiovascular disease in subsequent years.
