# Erratum, Vol. 16, April 18 Release

**DOI:** 10.5888/pcd16.180308e

**Published:** 2019-05-09

In the article “Association Between Changes in Postpartum Weight and Waist Circumference and Changes in Cardiometabolic Risk Factors Among Women With Recent Gestational Diabetes,” the footnote was missing from Table 3 because of a technical error. The correction was made to our website on April 24, 2019, and appears online at https://www.cdc.gov/pcd/issues/2019/18_0308.htm. We regret any confusion or inconvenience this error may have caused.

